# Study on Influencing Factors of Construction Workers’ Unsafe Behavior Based on Text Mining

**DOI:** 10.3389/fpsyg.2022.886390

**Published:** 2022-04-19

**Authors:** Ping Li, Youshi He, Zhengguang Li

**Affiliations:** ^1^School of Management, Jiangsu University, Zhenjiang, China; ^2^School of Economics and Management, Yancheng Institute of Technology, Yancheng, China

**Keywords:** text mining, unsafe behavior, influencing factors, construction workers, topic model, network analysis

## Abstract

The unsafe behavior of construction workers is the key cause of safety accidents. The accident investigation report contains rich experience and lessons, which can be used to prevent and reduce the occurrence of safety accidents. In order to draw lessons from the accident and realize knowledge sharing and reuse, this paper uses text mining technology to analyze the data of 500 construction accident investigation reports in Shenzhen, China. Firstly, a Latent Dirichlet Allocation (LDA) topic model is used to identify the unsafe behavior of construction workers and its influencing factors. Then, with the help of Social Network Analysis, the importance of influencing factors and the relationship between them are identified. The results show that weak safety awareness, operating regulations, supervision dereliction of duty, equipment resources, and inadequate supervision of the construction party are the key and important factors. It is also found that there are correlations between weak safety awareness and supervision dereliction of duty, between equipment resources and poor construction environment, between organization and coordination and inadequate supervision of the construction party, and between operating regulations and hidden dangers investigation. This study not only helps to improve the theoretical system in the field of construction workers’ unsafe behavior but also helps managers to find the key control direction of construction safety, so as to effectively curb unsafe behavior of construction workers and improve the level of safety management.

## Introduction

Future prospects of the global construction industry continue to be promising. Despite the coronavirus epidemic in 2019, the global investment in energy efficiency in the construction industry has reached an unprecedented 11.4% in 2020, increasing from the United States $165 billion in 2019 to about United States $184 billion (IEA, 2021). In China, with the continuous acceleration of urbanization in recent years, the construction industry has achieved unprecedented development. In 2020, the total added value of the construction industry was 7,299.6 billion Yuan, an increase of 3.5% over the previous year (National Bureau of Statistics of China, 2021). The rapid development of the construction industry has not only greatly promoted the growth of the national economy but also promoted the social labor and employment. However, the construction industry has the characteristics of a long construction cycle, complex working environment, and strong personnel mobility. It is a typical industry with a high incidence of safety accidents (Ji et al., 2021). Taking the production safety accidents of housing and municipal engineering as an example, the latest statistics of national engineering quality and safety supervision information platform of the Ministry of Housing and Urban-Rural Development of the People’s Republic of China show that in 2021, the number of production safety accidents of housing and municipal engineering in China has reached to 721, with 803 deaths, an increase in 29 accidents and 8 deaths over the previous year, all of which have increased to varying degrees. Frequent construction safety accidents have not only brought huge economic losses but also caused a strong negative social impact, which has seriously affected the high-quality development of the construction industry. Therefore, the control and prevention of construction production safety accidents have attracted much national attention.

Construction production workers are front line personnel of construction projects. They are not only the direct victims of safety accidents but also their unsafe behavior is the key cause of construction safety accidents (Yu et al., 2017). Studies have shown that more than 80% of safety production accidents in the construction industry are closely related to the unsafe behavior of construction workers (Yang et al., 2021). Therefore, from the key element of “human” in the construction system as the starting point, it is very necessary to accurately identify the influencing factors of construction workers’ unsafe behavior and explore the interaction between various factors, so as to reduce or avoid unsafe behavior of construction workers and control the occurrence of safety accidents.

At present, academia pays extensive attention to the influencing factors of construction workers’ unsafe behavior. Scholars mainly analyze the influencing factors of construction workers’ unsafe behavior from following four aspects: human, material, management, and environment. For example, psychological capital (Eid et al., 2012), physiology (Yang et al., 2021), safety awareness and attitude (Mohajeri et al., 2021; Liang et al., 2022), and work experience (Alizadeh et al., 2015; Rey-Merchán et al., 2021) are human factors; personal protective equipment (Amiri et al., 2014) and construction equipment (Castillo-Rosa et al., 2017) are material factors; safety promotion policy (Man et al., 2021), safety training (Man et al., 2021), safety atmosphere (Liao et al., 2014), safety supervision (Fang et al., 2015), safety management, and safety culture (Wang et al., 2016; Asilian-Mahabadi et al., 2018) are management factors; and working environment (Harsini et al., 2021) and social environment (Aven and Renn, 2009; Ma et al., 2021) are environmental factors. However, these studies only focus on the explicit analysis of the influencing factors of construction workers’ unsafe behavior, and few studies have focused on the implicit correlation analysis between the influencing factors. At the same time, due to the limitations of the development of tools and means, the utilization of data is not sufficient. With the rapid development of Natural Language Processing (NLP), unstructured building document data can also be transformed into structured information through NLP to realize the automatic analysis of tacit knowledge in the content of construction documents, so as to realize efficient risk management (Zhang et al., 2019; Baker et al., 2020; Wu et al., 2022).

At present, the degree of information disclosure on Chinese government websites at all levels has been continuously improved, and the information module in key areas has provided a large number of construction safety accident investigation reports that contain construction safety accident case information. As China’s Special Economic Zone, national economic center city and international city, Shenzhen, China, has a leading position in the disclosure of government website information in the country. According to the Blue Book “China government transparency index report” issued by the Chinese Academy of Social Sciences in recent years, since 2017, the transparency index of Shenzhen municipal government has ranked first, third, and second among the larger municipal governments, especially in 2018, 2019, and 2020. The information disclosure level of the Shenzhen municipal government website is high, and its key area information disclosure module provides a large number of work safety investigation reports. The accident investigation report is a legal document that reflects the real situation of the accident and puts forward handling opinions in the accident investigation (State Council of the PRC, 2007). The report gives an objective and true description of the process and causes of the accident, which provides an objective basis for the safety research of construction accidents. Through the analysis of the accident investigation reports, researchers can obtain the authoritative data related to the accidents, systematically analyze the unstructured data, and correlate the various factors related to construction safety, so as to identify the potential causes of construction workers’ unsafe behavior.

Text mining is a process of extracting valuable information or knowledge from a large number of text data. The methods used in text mining include information extraction, topic tracking, summarization, categorization, clustering, concept linkage, and information visualization (Gupta and Lehal, 2009). The Latent Dirichlet Allocation (LDA) model is a topic model widely used in the field of text mining (Blei et al., 2003). It can effectively extract implicit topics from large-scale documents and corpora. The model is particularly ideal for long text topic mining (Jin et al., 2010; Chou et al., 2017) and has a good adaptability in topic discovery (Zhu et al., 2016), topic evolution (Zhu et al., 2016; Xie et al., 2020), and topic tracking (Yeh et al., 2016; Zhang et al., 2017). Social Network Analysis is a quantitative analysis tool evolved from network theory and integrated with mathematical methods and graph theory (Scott, 1988). Compared with other research methods, it can not only reflect the position of individuals in the whole but can also show the interdependence between individuals (Serrat, 2017). This method can use large-scale network text data mining to obtain virtual relational structure data and has good plasticity in information dissemination and content interaction (Dunne, 2012; Smith, 2013).

In view of this, this paper selects 500 construction production accident investigation reports in Shenzhen, China from 2017 to 2021 as data samples, uses the LDA model to identify the influencing factors of construction workers’ unsafe behavior, and further explores the importance of influencing factors and the correlation between factors by using Social Network Analysis technology. The purpose of the study was to provide a basis for standardizing the operation behavior of construction workers, so as to reduce the occurrence of construction workers’ unsafe behavior and the incidence of construction safety accidents.

This study has the following contributions: first, text mining technology is used to analyze the influencing factors of construction workers’ unsafe behavior, which provides a new idea for the study of scientific management of construction safety in the era of big data. Second, the in-depth study on the influencing factors of construction workers’ unsafe behavior not only helps managers to trace the root causes of unsafe behavior but also expands the existing literature in the field of construction workers’ unsafe behavior.

## Methodology

### Overview of Text Mining Model Framework

**FIGURE 1:**
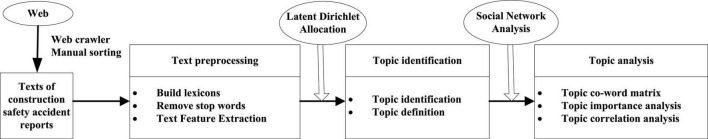
The framework of the text mining model.

The process of text mining includes text preprocessing, structured data, data analysis, result visualization, knowledge discovery, and other steps. The text mining model of influencing factors of construction workers’ unsafe behavior, which is constructed based on the text mining process, is shown in Figure 1. The model takes the construction safety accident investigation report as the text mining corpus. Firstly, an LDA topic model was used to mine the topics of construction workers’ unsafe behavior and its influencing factors. Then, on this basis, Social Network Analysis was used to construct the topic co-occurrence network of construction workers’ unsafe behavior and its influencing factors, so as to identify the importance and correlation of the influencing factors.

### Topic Model of Construction Workers’ Unsafe Behavior and Its Influencing Factors

The accident causing theory points out that the movement of people and things is carried out in a certain environment, and the unsafe behavior of people should be combined with other factors (unsafe state of things, environment, etc.) (Hopkins, 2001). In the accident report, the process and causes of the accident are described in detail, objectively and truly, and the accident safety is systematically and comprehensively analyzed from the aspects of human, material, environment, and management. Table 1 classifies some accident reports in terms of unsafe behavior and human, material, environmental, and management factors (Amiri et al., 2014; Li et al., 2015; Castillo-Rosa et al., 2017; Qiao et al., 2018; Harsini et al., 2021; Malakoutikhah et al., 2021; Man et al., 2021; Yang et al., 2021). Therefore, the construction accident investigation report can become an important basis for the analysis of construction workers’ unsafe behavior and its influencing factors.

**TABLE 1:** Analysis of accident statistical items.

Items	Report description (part)	References
Unsafe behavior	• Construction workers enter the dangerous area of construction for risky work • Failing to wear labor protection articles as required during operation	Qiao et al., 2018
Human factors	• Weak safety awareness, violation of the company’s safety operation management regulations • Older, poor physical condition, suffering from arteriosclerosis, fatty liver, cervical, thoracic and lumbar spine and other diseases	Man et al., 2021; Yang et al., 2021
Material factors	• The trampled self-made horse stool does not meet the safety regulations • Failing to distribute labor protection articles to workers as required	Amiri et al., 2014; Castillo-Rosa et al., 2017
Environmental factors	• The elevator derrick has not taken such sealing measures as compartment hard protection (formwork and scaffold board closed) and soft protection (bag net closed) • No obvious safety warning signs have been set at the edge of the foundation pit of the bearing platform of the brick matrix foundation	Harsini et al., 2021; Malakoutikhah et al., 2021
Management factors	• Failure to carry out safety education and training for construction personnel • Failure to supervise and urge the implementation of construction safety management	Li et al., 2015; Man et al., 2021
		

In view of the relatively long text of the accident investigation report, this paper has used the classic LDA topic model to mine construction workers’ unsafe behavior and its influencing factors. The basic assumption of the LDA model is that each document is composed of a mixture of topics with a certain probability, and each topic is also composed of a mixture of feature words with a certain probability, so as to form a three-layer Bayesian probability model of “document topic feature words.”

The LDA topic model needs to preset the number of topics, Blei et al. (2003) used confusion to determine the optimal number of topics K. Confusion degree is a commonly used evaluation index in the statistical language model. It refers to the reciprocal of the geometric mean of the similarity of each sentence contained in the corpus. The generalization ability of the model increases with the decrease of confusion degree. The calculation formula of confusion is:


(1) $$P\,e\,r\,p\,l\,e\,x\,i\,t\,y=\exp\left\{-\frac{\sum_{\text{d}=1}^{M}\log p\,\left(w_{d}\right)}{\sum_{d=1}^{M}N_{d}}\right\}$$

Where, _*p(w_d_)*_ refers to the probability of each word in the test set and _N_d__ represents the total number of all words in the test set. The lower confusion score reveals the higher prediction ability of the model.

### Topic Relationship Model of Construction Workers’ Unsafe Behavior and Its Influencing Factors

The theory of trajectory intersection between man and machine is a theory of accident causes, which points out that the intersection of human trajectory and object trajectory will constitute an accident. In many cases, people and things are mutually causal, and sometimes the unsafe state of things will also induce people’s unsafe behavior (Zhai, 2013). Although the LDA topic model can help to identify the topic of text, it is difficult to identify the relationship between topics in fine-grained by topic mining alone. A social network is a collection of social actors as nodes and their relationships (Freeman, 2004). The actor is the topic, and relationship refers to the number of times that two topics appear together in the text. The introduction of Social Network Analysis can clearly show the relationship network between topics and provide support for the analysis of the importance of topic words, network status, and related words. Therefore, based on the above analysis results of the LDA topic model, this paper introduces Social Network Analysis to identify the core influencing factors of construction workers’ unsafe behavior and the relationship between influencing factors.

Firstly, the topic co-occurrence matrix is constructed. Summarize the subject words generated by the LDA model and build a co-occurrence network.


$$T=\left[\begin{array}{ccc} a_{11} & \ldots & a_{1\,\text{i}}\\ \vdots & \ddots & \vdots \\ a_{i\,1} & \ldots & a_{i\,i} \end{array}\right]$$

Where, the element *_a_ii__* on the diagonal of the subject co-occurrence matrix is 0; the element _*a_ik_*(0<*i*<*k*)_ on the non-diagonal line is the number of times two topics appear in the same text. The greater the value, the stronger the correlation between the two topics.

Secondly, it measures the importance of the topic. Degree centrality emphasizes the individual value of a node and measures the importance of the node in the whole network. The higher the value, the more nodes related to the point, the more they are in the center of the network, the more resources they occupy, and it can also reflect the control effect of the point on the whole network. The measure of degree centrality usually uses indicators, such as degree centrality and relative degree centrality.

Degree centrality is a measure of the differentiation of network nodes, and its calculation formula is:


(2) $${\text{C}}_{D}\left(n_{i}\right)=d\,\left(n_{i}\right)$$

The relative degree centrality is the ratio of the actual degree to the maximum possible degree, and its calculation formula is:


(3) $$C_{D}^{{}^{*}}=\frac{d\,\left(n_{i}\right)}{\text{n}-1}$$

In Equations 2, 3, _C_*D*__(*ni*)__ represents the absolute centrality of node _i_, _*C*_D_^*^_ represents the relative centrality of node _i_, _n_represents the number of nodes, and _*d(n_i_)*_ represents the number of direct connections between node i and other nodes.

Finally, it identifies the relevance of the topic. Co-cohesive subgroup analysis is an important method in Social Network Analysis. Its purpose is to reveal the actual or potential relationship between social actors. When the relationship between some actors in the network is so close that they are combined into a sub-group, such a group is a cohesive subgroup. This paper uses the convergence of iterated correlations (CONCOR) method in UCINET software to analyze the influencing factors of construction workers’ unsafe behavior and then identifies the correlation between the influencing factors.

## Data Analysis and Results

### Data Material Collection

The safety accident investigation report of this study comes from the key information disclosure module of Futian District, Luohu District, Yantian District, Nanshan District, Baoan District, Longgang District, Longhua District, Pingshan District, Guangming District, and Dapeng New Area government website in Shenzhen, China. Firstly, this paper has designed a Python crawler program, sent an Hypertext Transfer Protocol (HTTP) request through the request module to obtain the HTML page of the accident investigation report module of 10 district government websites in Shenzhen, parsed the HTML formatted content with the beautifulsoup4 module, and obtained 1,024 information data of the investigation report, such as title, time, and content links (the deadline was 20 January 2022). On this basis, a total of 500 construction safety accident investigation reports from 2017 to 2021 were selected as the data used for text mining in this paper. The number of construction safety accident reports used for text mining in various districts of Shenzhen from 2017 to 2021 is shown in Table 2.

**TABLE 2:** Statistics of the number of texts in each district of Shenzhen from 2017 to 2021.

No.	District	Numbers of texts	No.	District	Numbers of texts
1	Futian	35	6	Longgang	99
2	Luohu	22	7	Longhua	79
3	Yantian	14	8	Pingshan	26
4	Nanshan	47	9	Guangming	46
5	Bao’an	119	10	Dapeng New Area	13

Due to the long length of the safety accident investigation report, in order to reduce the impact of phrases unrelated to the study of construction safety workers’ behavior and its influencing factors on the excavation results, only the contents of “accident process,” “accident cause,” and “responsibility determination” in the investigation report were selected as the corpus of text excavation. Manually revised the contents and formats of these reports and summarized them into a TXT text file. Each investigation report is a line in the text, a total of 500 lines of data, forming a corpus to be mined.

### Data Preprocessing

Firstly, the text mining corpus was segmented. In order to improve the efficiency of word segmentation and ensure the accuracy and integrity of word segmentation, this paper has adopted the stop word list of Harbin Institute of Technology, Baidu stop word list, and the stop word library of machine dictionary Intelligent Laboratory of Sichuan University, and on this basis, it has used those useless high-frequency words that were less helpful to the interpretation of the results, such as “limited liability company,” “unit,” “Shenzhen,” and others joined the stop word dictionary to build a user-defined stop word dictionary. This paper has used the jieba library of Python language to complete the word segmentation of the text mining corpus. Then, a text mining corpus was constructed to form a “document word” matrix. Since the LDA model uses a bag of words (BOWs) to generate a word frequency vector (Zhong et al., 2020), this paper has used CountVectorizer function in the sklearn library to realize the “document word” matrix.

### Latent Dirichlet Allocation Topic Model Analysis

The LDA topic model will generate a “topic word” matrix and a “document topic” matrix with a certain probability according to the “document word matrix”, so as to screen out some topics. This paper has used Python language, sklearn library, and pyLDAvis library for topic generation and visualization.

#### Determination of Topic Quantity

**FIGURE 2:**
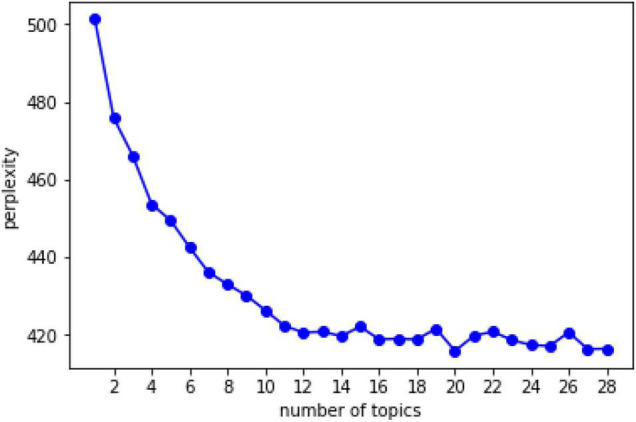
Topic confusion value diagram.

This paper has mainly made exploratory analysis based on the LDA model and has not set the expected results according to general cognition. Therefore, the confusion degree method was used to determine the number of topics, as shown in Figure 2.

As can be seen from Figure 2, with the increasing number of topics, the degree of confusion decreases rapidly and tends to be flat when the number of topics is 12. According to Equation 1, the lower the degree of confusion a model has, the stronger the representation ability of the model. However, in the LDA model, the more the number of topics, the lower the degree of confusion. In order to avoid over fitting caused by taking the confusion degree as the index, Blei et al. (2003) used the confusion degree subject number curve to measure the model representation ability. When the curve tends to be flat, it shows that the marginal effect of increasing the number of topics is very small. Take the inflection point as the number of topics, and there is no need to increase the number of topics excessively. Therefore, the final number of topics was 12.

#### Topic Description and Visualization

In this paper, the Latent Dirichlet Allocation function of the sklearn library was used to train the LDA model, and the topic extraction of construction workers’ unsafe behavior and its influencing factors was carried out on the text mining corpus. Where, the number of topics K was set to 12, α was set to 1/K, β is set to 0.01, and the number of iterations was 1,000.

Because there are many feature words extracted by the LDA model and too many topic feature words are difficult to be directly used in the practical analysis, this study has selected the top 8 words as topic representatives and then carried out topic feature recognition and induction. According to the topic, characteristic words found by the LDA model and combined with the analysis of accident statistical items in Table 1, the labels of these 12 topics are manually defined and finally summarized in Table 3.

**TABLE 3:** Topic extraction results of construction workers’ unsafe behavior and its influencing factors.

No.	Keywords	Behavior/influencing factors	Topic description
1	Supervision, project, responsibility, matters involved, performance, inspection, person in charge, liability	Management factors	Inadequate supervision of the construction party (SF1)
2	Hoisting, crane, steel wire rope, ground, use, fracture, falling, working at height	Material factors	Equipment resources (MF1)
3	Implement, responsibility, operation, inspection, violation, operating procedures, hazards, management systems	Management factors	Operating regulations (SF2)
4	Safety, management, awareness, use, labor, incident, inspection, no	Human factors	Weak safety awareness (HF1)
5	Performance, inspection, supervision, overall, no, problem, inspect, responsibility	Management factors	Supervision dereliction of duty (SF3)
6	Demolition, wall, bottom, method, use, labor, excavation, violation	Unsafe behavior	Construction depend on experience (B1)
7	Protective equipment, failure to, wear, use, scaffold, safety belt, performance, violation	Unsafe behavior	Protective equipment not worn (B2)
8	Live, wire, conductor, exposed, leakage, current, temporary, wiring	Environmental factors	Poor construction environment (EF1)
9	Organization, coordination, implementation, operation process, requirements, performance, failure, command	Management factors	Organization and coordination (SF4)
10	Movement, guardrail, climbing, instability, extrusion, get over, process, adventure	Unsafe behavior	Risk taking behavior (B3)
11	Qualification, have, contract awarding, acquisition, conditions, construction team, individual, illegal	Human factors	Construction qualification (HF2)
12	Discovery, troubleshooting, deficiency, hidden danger, analysis, dangerous, implementation, arrangement	Management factors	Hidden dangers investigation (SF5)

**FIGURE 3:**
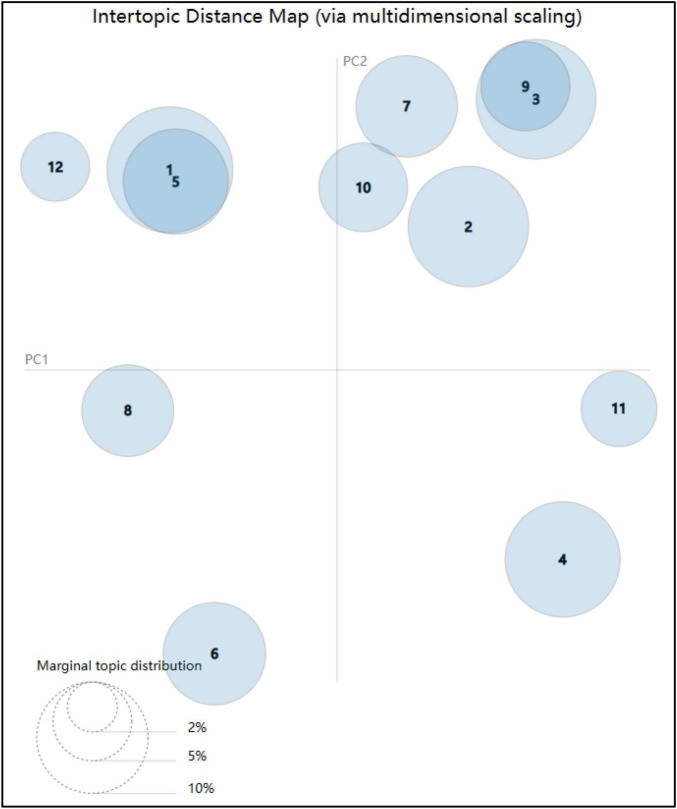
Topic visualization.

From the extracted topics, it can be seen that the calculation results of the LDA model include construction workers’ unsafe behavior and its influencing factors. Finally, the pyLDAvis library is used to visualize the extracted topics, and the visualization results are shown in Figure 3.

Different circles in Figure 3 represent different topics, and the numbers in the circles correspond to the topic serial number in Table 3. The distance between circles is used to describe the distance between topics, that is, to express the proximity between topics. There is overlap in the two circles, indicating that the characteristic words in the two topics are crossed. For example, the overlapping area of the circle between Topics 1 and 5 is relatively large, mainly because Topic 1 is related to the adverse supervision of the construction party and Topic 5 is related to the dereliction of duty of the supervisor. Both topics have common characteristic words, such as “inspection” and “responsibility.”

### Topic Social Network Analysis

#### Co-occurrence Network of Construction Workers’ Unsafe Behavior and Its Influencing Factors

In this part, firstly, the characteristic words of LDA were sorted and summarized according to Table 3, and the characteristic words under the same subject were used as the subject characteristic identification to construct the subject co-occurrence matrix, as shown in Table 4. In the co-occurrence matrix, the diagonal element is 0, and the non-diagonal element is the number of times that two topics appear simultaneously in 500 accident investigation report texts. For example, B1 (construction depends on experience) and HF1 (weak safety awareness) appeared 156 times at the same time.

**TABLE 4:** Topic co-occurrence matrix.

	B1	B2	B3	HF1	HF2	SF1	SF2	SF3	SF4	SF5	MF1	EF1
B1	0	51	68	156	81	92	78	54	16	36	52	17
B2	51	0	121	168	36	87	65	76	57	25	45	27
B3	68	121	0	147	89	78	81	89	79	17	36	21
HF1	156	168	147	0	18	16	96	20	31	29	23	19
HF2	81	36	89	18	0	43	36	19	12	45	16	43
SF1	92	87	78	16	43	0	15	21	53	18	32	31
SF2	78	65	81	96	36	15	0	101	19	13	85	54
SF3	54	76	89	20	19	21	101	0	14	51	75	84
SF4	16	57	79	31	12	53	19	14	0	45	67	36
SF5	36	20	17	29	45	18	13	51	45	0	103	56
MF1	52	45	36	23	16	32	85	75	67	103	0	12
EF1	17	27	21	19	43	31	54	84	36	56	12	0
												

**FIGURE 4:**
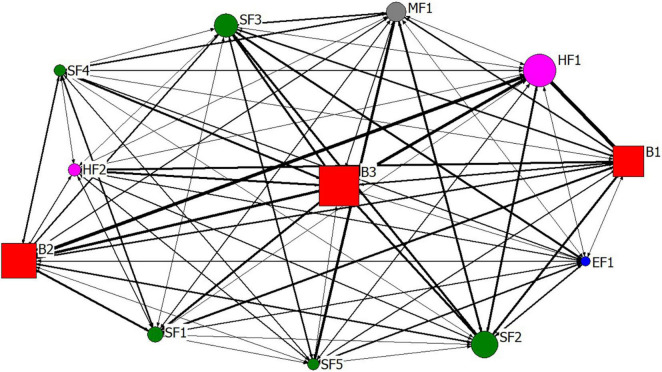
Co-occurrence network of construction workers’ unsafe behavior and its influencing factors.

Based on the topic co-occurrence matrix, this paper has used UCINET software to draw the co-occurrence network diagram of unsafe behavior of construction workers and its influencing factors, as shown in Figure 4. In Figure 4, different nodes of the network represent different topics, in which the square represents the topic of construction workers’ unsafe behavior, the circle represents the topic of influencing factors of unsafe behavior, and the co-occurrence relationship of each topic is represented by the connection of nodes. The size of the circle (square) represents the importance of the topic in the co-occurrence network, and the size between the two nodes represents the closeness of the two topics in the co-occurrence network. For example, among all circles, circle HF1 (weak safety awareness) has the largest circle, indicating that the topic is the most important of all influencing factor topics. Among all node connections, HF1 (weak safety awareness) has the thickest connection with blocks B1 (Construction depend on experience) and B2 (protective equipment not worn), indicating that this influencing factor is most closely related to the unsafe behavior, such as construction depends on experience and protective equipment not worn.

#### Analysis on the Importance of Unsafe Construction Workers’ Influencing Factors

Point centrality is a key index to reveal the importance of nodes in social networks. If the node is in the center of the network, its point centrality value will be the highest. The importance of the influencing factors of construction workers’ unsafe behavior can be revealed by point to the center analysis of the influencing factors of construction workers’ unsafe behavior. Table 5 shows the score and ranking of point centrality of influencing factors of construction workers’ unsafe behavior, where Degree represents absolute centrality, NrmDegree represents standardized centrality, and Share represents the ratio of the centrality of each node to total centrality. According to the degree value, HF1 (weak safety awareness) ranks first among all influencing factors, indicating that this topic has the most important impact on the unsafe behavior of construction workers.

**TABLE 5:** Point centrality score of influencing factors of construction workers’ unsafe behavior.

No.	Factor	Degree	NrmDegree	Share
1	HF1	723.000	39.123	0.104
2	SF2	643.000	34.794	0.092
3	SF3	604.000	32.684	0.087
4	MF1	546.000	29.545	0.078
5	SF1	486.000	26.299	0.070
6	HF2	438.000	23.701	0.063
7	SF5	433.000	23.431	0.062
8	SF4	429.000	23.214	0.061
9	EF1	400.000	21.645	0.057
				

According to the share value, this paper uses the following standards to classify the influencing factors of construction workers’ unsafe behavior: Share ≥ 0.08 is the key factor; 0.07 ≤ Share < 0.08 is an important factor; the factor of 0.07 ≤ Share ≤ 0.06 is the secondary factor; the factors with Share less than 0.06 are general factors. See Table 6 for details.

**TABLE 6:** Identification of influencing factors of construction workers’ unsafe behavior.

Grade	Influencing factors
Key factor	Weak safety awareness (HF1)
	Operating regulations (SF2)
	Supervision dereliction of duty (SF3)
Important factor	Equipment resources (MF1)
	Inadequate supervision of the construction party (SF1)
Secondary factor	Construction qualification (HF2)
	Hidden dangers investigation (SF5)
	Organization and coordination (SF4)
General factor	Poor construction environment (EF1)
	

#### Correlation Identification of Influencing Factors of Construction Workers’ Unsafe Behavior

**FIGURE 5:**
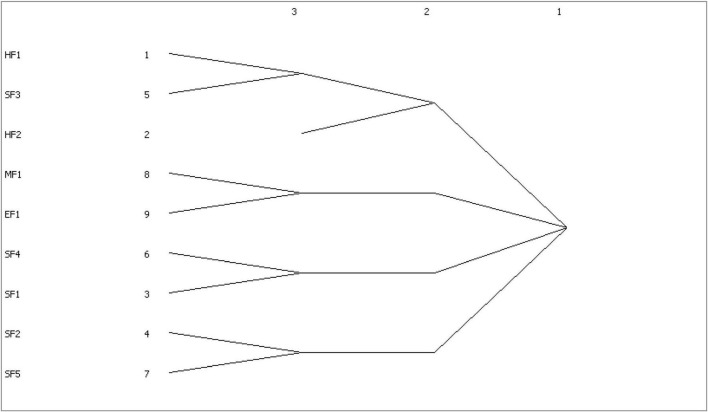
Network aggregation subgroup tree of influencing factors of construction workers’ unsafe behavior.

A condensed subgroup describes a subset with relatively strong, direct, close, and frequent connections. The cohesive subgroup analysis shows that the substructure of the influencing factors network of construction workers’ unsafe behavior is closely related. As can be seen from Figure 5 that node HF2 alone constitutes a cohesive subgroup, while nodes HF1 (weak safety awareness) and SF3 (supervision dereliction of duty), MF1 (equipment resources) and EF1 (poor construction environment), SF4 (organization and coordination) and SF1 (inadequate supervision of the construction party), SF2 (operating regulations), and SF5 (hidden dangers investigation) have a strong trust relationship, and several small groups have been formed.

## Conclusion and Future Work

Based on the accident investigation reports on the websites of the people’s governments of various districts in Shenzhen, China from 2017 to 2021, this paper uses the LDA topic model to identify construction workers’ unsafe behavior and its influencing factors from the accident reports and constructs the topic social relationship network to identify and analyze the importance of the influencing factors of construction workers’ unsafe behavior and the correlation between the influencing factors. The main conclusions are as follows:

(1) With the help of an LDA topic model, three kinds of construction workers’ unsafe behavior and nine influencing factors are identified from the total accident report of construction. The three kinds of construction workers’ unsafe behavior is a construction that depends on experience, protective equipment not being worn and risk-taking behavior. The nine influencing factors of construction workers’ unsafe behavior are inadequate supervision of the construction party, equipment resources, operating regulations, weak safety awareness, supervision dereliction of duty, poor construction environment, organization and coordination, construction qualification, and hidden dangers investigation. Among them, weak safety awareness and construction qualification are human factors; equipment resources are material factors; inadequate supervision of the construction party, operating regulations, supervision dereliction of duty, organization and coordination, and hidden dangers investigation are management factors; the poor construction environment is environmental factors.
(2) The topic co-occurrence network is used to show the co-occurrence relationship between construction workers’ unsafe behavior and its influencing factors, and the nine influencing factors are divided into four levels, i.e., key factors, important factors, secondary factors, and general factors, according to the centrality. Among them, weak safety awareness, operating regulations, supervision dereliction of duty, equipment resources, and inadequate supervision of the construction party are the key and important factors, and the managers should attach great importance to them and focus on control.
(3) Through the agglomerative subgroup analysis of the influencing factors of construction workers’ unsafe behavior, we can see the internal relationship of the influencing factors. According to Figure 5, it can be found that there are correlations between weak safety awareness and supervision dereliction of duty, between equipment resources and poor construction environment, between organization and coordination and inadequate supervision of the construction party, and between operating regulations and hidden dangers investigation. It can be seen that the effects of some influencing factors on construction workers’ unsafe behavior are superimposed. Managers should systematically analyze them and put forward targeted measures.

The conclusions of this paper have important theoretical and practical significance. First, this paper uses text mining technology to explore the influencing factors of construction workers’ unsafe behavior, which provides a new idea for the study of scientific management of construction safety in the era of big data. Second, this study not only finds the key and important factors of construction workers’ unsafe behavior but also finds the implicit correlation of the influencing factors, thus broadening the research boundary of the influencing factors of construction workers’ unsafe behavior. Third, the findings of this paper will help regulators to systematically examine the motivations of construction workers’ unsafe behavior and then formulate targeted measures to reduce construction workers’ unsafe behavior and incidence of construction safety accidents.

The limitations of this paper need to be improved in future research. Since this study only uses the accident investigation reports in Shenzhen, China as the data sample, the research data are limited and have a certain regionality, and the generalizability of the conclusions of this study cannot be guaranteed. In the follow-up study, the number and regional scope of samples can be appropriately expanded. In addition, this study only identifies the influencing factors of workers’ unsafe behavior and their importance and relevance by using text mining technology. On this basis, subsequent studies can further explore the impact path of various influencing factors on construction workers’ unsafe behavior.

## Data Availability Statement

The original contributions presented in the study are included in the article/supplementary material, further inquiries can be directed to the corresponding author/s.

## Ethics Statement

Ethical review and approval was not required for the study on human participants in accordance with the local legislation and institutional requirements. Written informed consent from the (patients/participants or patients/participants legal guardian/next of kin) was not required to participate in this study in accordance with the national legislation and the institutional requirements.

## Author Contributions

PL contributed to methodology, data analysis, and writing. YH contributed to conception and methodology. ZL contributed to writing and checking. All authors contributed to the article and approved the submitted version.

## Conflict of Interest

The authors declare that the research was conducted in the absence of any commercial or financial relationships that could be construed as a potential conflict of interest.

## Publisher’s Note

All claims expressed in this article are solely those of the authors and do not necessarily represent those of their affiliated organizations, or those of the publisher, the editors and the reviewers. Any product that may be evaluated in this article, or claim that may be made by its manufacturer, is not guaranteed or endorsed by the publisher.
